# A fast region of interest algorithm for efficient data compression and improved peak detection in high-resolution mass spectrometry

**DOI:** 10.1007/s00216-024-05718-7

**Published:** 2025-01-09

**Authors:** Oskar Munk Kronik, Jan H. Christensen, Nikoline Juul Nielsen, Selina Tisler, Giorgio Tomasi

**Affiliations:** Department of Plant and Environmental Science, University of Copenhagen, Thorvaldsensvej 40, DK-1871 Frederiksberg, Denmark

**Keywords:** Non-target screening, Data preprocessing, Region of interest, Objective parameterisation, High-resolution mass spectrometry, Chromatography

## Abstract

Liquid chromatography coupled to high-resolution mass spectrometry (LC-HRMS) is commonly used for identification of compounds in complex samples due to the high chromatographic and mass spectral resolution provided. In subsequent data processing workflows, it is imperative to preserve this resolution to fully exploit the data. “Region of interest” (ROI) algorithms were introduced as a better alternative to equidistant binning for compressing HRMS data because they better preserve the mass spectral resolution. In this paper, we present a new ROI algorithm that improves on the selection of contiguous *m*/*z* traces, amongst others by introducing the concept of chromatographic filter, allows for an automated approach to optimise the admissible mass-to-charge deviation (*δ*_*m/z*_) and can be used to match ROIs across multiple samples. The algorithm was tested on a LC-HRMS dataset comprised of 21 replicate injections of a wastewater effluent extract and assessed on its ability to correctly retrieve the ROI’s relative to 57 compounds and match them across all injections. In summary, it achieved a ten-fold compression rate in on-disk storage at a noise threshold of 200 counts, and the median ROI length matched the observed chromatographic peak width (12–23 points). Correct ROI matching with a mass accuracy of 9 ppm was observed for 52 compounds across all 21 injections with only one compound split between two adjacent *m/z* traces in six runs. Overall, the new algorithm performed favourably compared to the ROI algorithm currently used in the well-established ROI-MCR (multivariate curve resolution) workflow for deconvolution of HRMS chromatographic data.

**Supplementary Information:**

The online version contains supplementary material available at 10.1007/s00216-024-05718-7.

## Introduction

High-resolution mass spectrometry (HRMS) is becoming increasingly common in target-, suspect and non-target screening (NTS) analysis of complex samples. Modern HRMS systems have led to a significant improvement in our ability to separate and identify known and unknown compounds because of their high mass spectral resolution with Δ mass-to-charge ratio (*m/z*) down to 0.001, and *m/z* deviation of a few parts per million (ppm) [1]. It is imperative to maintain this mass spectral resolution and low *m/z* deviation as this allows to estimate tentative molecular formula used as a first step in identification of non-target compounds [2]. The data files obtained from HRMS instruments quickly become large, and unwieldy, hence, it is not uncommon to have problems with exceeding the memory of the computer when processing multiple files at the same time [3]. Data compression is often used as a first step in HRMS data processing workflows as a solution to reduce the memory demand on the processing computer; however, the compression will inevitably sacrifice some of the mass spectral resolution of the data. Equidistant *m/z* binning has traditionally been used; however, it has been argued that this approach poses a significant risk of splitting mass spectral measurements from a single compound due to the rigid positioning of bin boundaries [3, 4]. Data compression algorithms are widely used in data science [3, 5]. Specialised approaches have been developed to exploit the unique characteristic of HRMS hyphenated with chromatography. One notable example is the *centWave* algorithm [6], which offers a flexible alternative to equidistant binning. Unlike traditional methods, *centWave* performs lossy compression by dynamically updating *m/z* boundaries along the chromatographic dimension, with the centre value determined by the data points included in the region of interest (ROI). Other innovative implementation utilises the data voids created around the centroid *m/z* when converting the continuum files to centroided files [7]. The use and implementation of a ROI algorithm prior to multivariate curve-resolution was pioneered by Tauler et al. [8]. Their ROI algorithm, which in this study will be referred to as the TGJ algorithm after the initials of its authors, has been shown to reduce the data size significantly whilst better preserving mass spectral resolution compared to equidistant binning of the *m/z* axis [9]. However, we have observed that the processing time of the TGJ algorithm can be up to several hours per sample with approx. 10^6^ data points, which is not uncommon for a liquid chromatography (LC)-HRMS analysis of 25 min, when using noise thresholds relevant for trace level analysis [7]. If the processing is not subjected to parallel processing, this would hinder their use for large datasets with more than a few samples, since the processing time would be excessively long. Pérez-López et al. [10] introduced a pre-processing step to filter out data with an undefined charge state, i.e. noise to reduce the data size, hence decrease the computational time of the subsequent ROI algorithm. Whilst improving on speed, it does not address the efficiency of the current algorithm. Therefore, there is still a need for improving the efficiency of the ROI algorithm in the computing environment MatLab, where a large body of the code base for curve-resolution and multilinear models is present, to enable its use for larger datasets [11–14].

In this work, a new ROI algorithm was developed and its performance tested with respect to its processing speed, data reduction capabilities, length of the ROIs detected, mass resolution and accuracy retained and the risk of splitting signals originating from one compound. The algorithm presented herein will from now on be referred to as the OMG algorithm, after the initials of the two authors who devised it. We compared the performance of the OMG algorithm to the existing MatLab-based TGJ ROI algorithm. The performance of the two algorithms were compared using a dataset consisting of 21 injections of a pooled wastewater effluent sample analysed using LC-HRMS. Furthermore, we present a novel and objective optimisation scheme for determining the admissible *m/z* deviation (*δ*_*m/z*_) of the ROI algorithm.

## Materials and methods

### Dataset and sample information

To compare the performances of the OMG and the TGJ algorithm, 21 LC-HRMS chromatograms originating from the same pooled wastewater effluent methanol extract were used. The dataset was used to validate the compound detection and matching capabilities of the ROI algorithm presented in this study. The methanol extracts were analysed at a relative enrichment factor of 50. The enrichment was achieved using solid-phase extraction. A detailed description of the sample set, the sample preparation protocol and the LC-HRMS method can be found in Tisler et al. [15].

For each LC-HRMS run, two mass spectral traces were obtained: a high and a low collision energy trace with correspondingly high and low degree of fragmentation, respectively. In this study, only the low energy trace was used since the aim of the study was to evaluate the speed performance, compound detection etc. For identification workflows, also the high energy trace should be used. In the GitHub repository where the algorithms are available (https://github.com/OskarMunkKronik/regionofinterest), there is a template file for importing and processing both the low and high energy traces for data obtained in data-independent acquisition (DIA). The data files were acquired in continuum mode and subsequently converted to centroid mode using the vendor software MassLynx™ (v 4.1, Waters, UK). The vendor format .Raw was converted to netCDF files using the vendor software Databridge (version 3.5 (NT), Micromass Ltd) to be able to process the data in MatLab (The MathWorks, Inc., USA, version R2022a). A list of 57 compounds previously identified through a non-target screening workflow by Tisler et al. [15] was used to compare the two algorithms. These compounds were present in the sample at the time of collection; i.e., they were not spiked in. Therefore, the dataset and problem can be considered representative of the task for future NTS workflows. The algorithms were run on a HP ProLiant DL380 Gen9 equipped with two Intel Xeon E5-2620v4 (2.1GHz, 8-core, 20MB Intel Smart Cache) and 192 GB of memory.

### The ROI algorithms

**Fig. 1:**
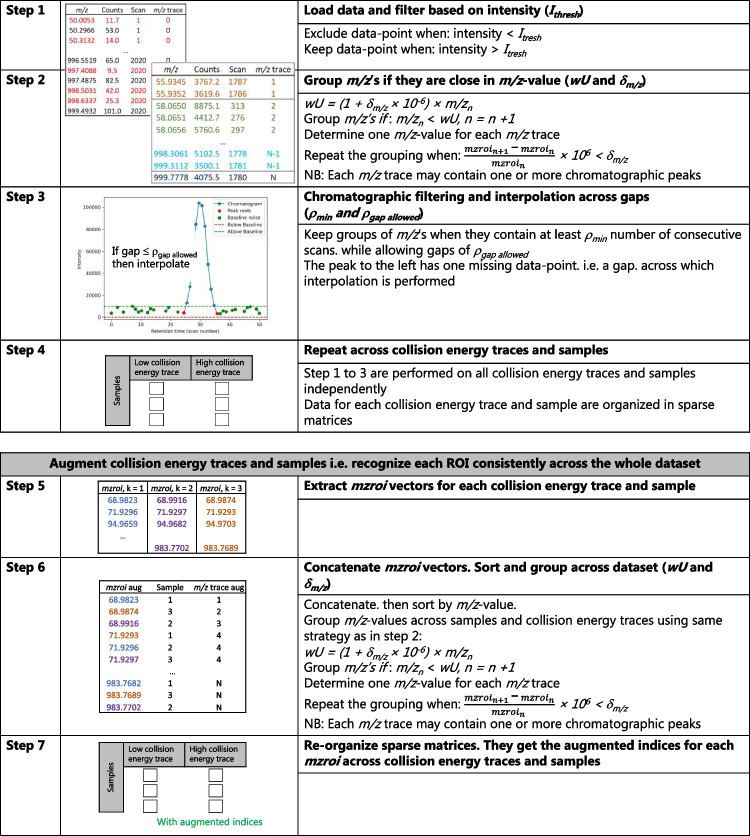
Flow chart of the developed ROI algorithm and the augmentation of samples and collision energy traces. The subscripts *n* denote the row index in the data matrix from steps 2 and 6, respectively, and *k* is the sample number

In the following, the acronym ROI will refer to a region confined both in the retention time and *m/z* dimensions, whereas the term *m/z* trace will be used to denote all the ROIs with *m/z*’s within the *δ*_*m/z*_ along the entire retention time dimension. In Fig. 1, the OMG algorithm is described in detail. The algorithm requires the choice of four parameters: (1) noise threshold (*I*_thresh_), (2) *δ*_*m/z*_, (3) a minimum number of consecutive scans (*ρ*_min_) within *δ*_*m/z*_ in order to be considered a ROI, *ρ*_gap allowed_, which allows for a specified number of missing value(s) inside a ROI, and across which data is interpolated. A preliminary visual inspection of the data indicated that the narrowest peaks in the chromatogram were eight scans wide; therefore, the same value was used for *ρ*_min_. In step 1 (Fig. 1), all the values with intensities < *I*_thresh_ (red numbers) are excluded. A higher *I*_thresh_ reduces algorithm processing time but risks omitting trace-level compounds. In step 2, the intensity-filtered matrix is sorted in ascending order by *m/z* values, enabling efficient grouping of *m/z* values less than an upper *m/z* limit (*wU*). See Eq 1:

1 $$wU=\left\{\begin{array}{cc}\left(1+\delta_{m/z}{\times10}^{-6}\right){\times\;m/z}_n,&i\mathrm f\delta_{m/z}\;\mathrm{is}\;\mathrm{in}\;\mathrm{ppm}\\{m/z}_n+\delta_{m/z},&i\mathrm f\delta_{m/z}\;\mathrm{is}\;\mathrm{in}\;\mathrm{Da}\end{array}\right.$$

When *n* is equal to 1, *δ*_*m/z*_ is multiplied by 0.5 since the first measured *m/z* value was expected to be the centre *m/z* of the first ROI. Whilst *m/z*_*n*_ < *wU*, the index number *n* of the data matrix is incrementally increased and these *m/z* values are grouped.

In step 3, an additional filter (henceforth referred to as the “chromatographic filter”) was implemented in the OMG algorithm to retain only relevant ROIs in the final dataset satisfying conditions 3 and 4, and filter out noise based on the following assumptions: Chromatographic peaks are expected to have consecutive measurements with a difference in their *m/z* values < *δ*_*m/z*_ (blue and red measurements in step 3, in Fig. 1), whereas electronic noise is assumed to be randomly distributed spike-events (e.g. single non-zero signals bracketed by zeros, green measurements in step 3 in Fig. 1). To accommodate for the case in which a single measurement within a chromatographic peak does not satisfy the requirements in step 3, the parameter *ρ*_gap allowed_ was introduced to minimise the risk of excluding such cases. In Fig. 1, an example of such a gap is shown inside the blue peak, and across which data will be interpolated if the *ρ*_gap allowed_ is set to ≥ 1.

The optimal value of *δ*_*m/z*_ was determined in the interval 0.01–0.1 Da as the one leading to the highest number of *m/z* traces detected, when calculated as an average of seven replicate injections. To provide input for curve resolution models or comparison across samples, the ROIs detected in different samples need to be augmented. An algorithm for such fusion processes has been developed and used herein to augment multiple samples and mass spectral traces when available. In short, it functions by concatenating the vectors of *m/z* values representative of a chromatographic peak or *m/z* trace (*mzroi*) for both the low- and high-collision energy trace for each sample (Fig. 1, step 6). Subsequently, this vector is sorted from lowest to highest *m/z* value and then the adjacent *m/z* values across samples and collision traces are checked as to if their *m/z* < *wU* (Fig. 1, step 6). When true, the *m/z* traces are grouped across samples and collision energies. These new groups are then subjected to the same iterative approach as shown in Fig. 1, step 2. The grouped *m/z* values of the traces from each sample and collision energy are used to calculate a new *m/z* value for each group of *m/z* trace in the augmented data matrix. The new *m/z* value is calculated as the median value of the included *m/z* traces or as the intensity weighted average of the included scan points. The latter can be used to minimise noise or baseline measurements influence on the calculated *m/z* value. This was used in this study. Therefore, if the same compound across different samples is grouped correctly, they will end in the same *m/z* trace and have identical *m/z* values in the augmented *mzroi*. The data augmentation algorithm uses the *m/z* information only. Therefore, each *m/z* trace can contain one or multiple peaks. The number of peaks in each *m/z* trace is sample dependent, where more peaks per *m/z* trace are expected if the number of isomers in a sample is large or precursor ions fragment into the fragment ions. The *MSroi* matrix (number of *m/z* traces × maximum scan number) of each sample is then organised according to their new indices in the augmented *mzroi* vector. The ROI algorithm can also generate a *MSroi* matrix in which each row in the *MSroi* from step 4 in Fig. 1 corresponds to a single ROI confined in the retention time and *m/z* dimension (i.e. one chromatographic peak or baseline segment) for feature-based workflows. The generated higher order tensors may subsequently be subjected to curve resolution modelling by either parallel factor analysis [14, 16] or sample-wise augmented multilinear curve-resolution [8, 16]. In such approaches, the data is segmented into smaller retention time windows, allowing the exclusion of the parts of *m/z* traces that contain only zeros across all samples. The algorithms are currently implemented in MatLab.

### Metrics for ROI algorithm comparison

The two ROI algorithms were compared based on their number of detected compounds and the *m/z* deviations obtained for the compounds previously identified by Tisler et al. [15]. The *m/z* deviation in ppm was calculated according to Eq. 2:

2 $$\mathrm m/\mathrm z\;\mathrm{deviation}=\frac{\mathrm m/{\mathrm z}_{\mathrm{observed}}-\mathrm m/{\mathrm z}_{\mathrm{true}}}{\mathrm m/{\mathrm z}_{\mathrm{true}}}\times10^6$$

where *m/z*_observed_ is the ROI extracted *m/z* for a given compound, and *m/z*_true_ is the expected *m/z* of the [M + H]^+^ ion. This was calculated for all the 57 compounds. The *m/z* trace detected by the ROI algorithm which was closest to the [M + H]^+^ ion of each of the 57 compounds was selected if three criteria were fulfilled: the *m/z* deviation < the given value of *δ*_*m/z*_, peak height > 10^4^ and retention time deviation < 0.2 min from the expected. This peak detection was applied to the raw data extraction using an *m/z* window of $\frac{{\delta }_{m/z}}{2}$. A maximum of 57 compounds were detected at *δ*_*m/z*_ = 0.10 Da and 52 at *δ*_*m/z*_ = 0.01 Da in the seven LC-HRMS chromatograms used for the optimisation. This was a subset of the 21 LC-HRMS chromatograms described in the “Dataset and sample information” section. An automated approach was used in this study to verify the correct augmentation of data matrices from individual samples. The root-mean-squared-*m/z* deviation (RMS-mDev) was calculated using Eq. 3:

3 $$\mathrm{RMS-mDev}=\sqrt{{\sum_{c=1}^{c=C }\frac{{\mathrm{(}\mathrm{m/z}}_{\mathrm{c},\mathrm{ observed}}-{\mathrm{m/z}}_{\mathrm{c,true}})}{{\mathrm{m/z}}_{\mathrm{c},\mathrm{true}}^{2}}}^{2}\times \frac{1}{C}}, \mathrm{for}\;s=1\dots S$$

where *s* denotes the sample ranging from 1 to *S*, and the known detected compound was denoted by *c* ranging from 1 to *C*. In this case, *S* was 21. The dimension of RMS-mDev was *S* × *1*.

For comparative purposes, the length of the ROIs detected by the TGJ algorithm was measured prior to interpolation across elution profile gaps. In the TGJ algorithm, potential gaps in the elution profiles are linearly interpolated between the two scan points bracketing the gap. Zero-centred, normally distributed random noise with a standard deviation of 0.3 × *I*_thresh_ is then added to all scan indices in the chromatogram. This approach also implies that negative intensities can be observed in the final data matrix and that the S/N can be significantly decreased for features with an already low S/N. The interpolation and addition of noise removes the inherent sparsity of the data altogether thereby increasing the algorithm’s memory footprint will increase.

## Results and discussion

### Optimisation scheme for determining the *m/z* deviation (*δ*_*m/z*_)

**Fig. 2:**
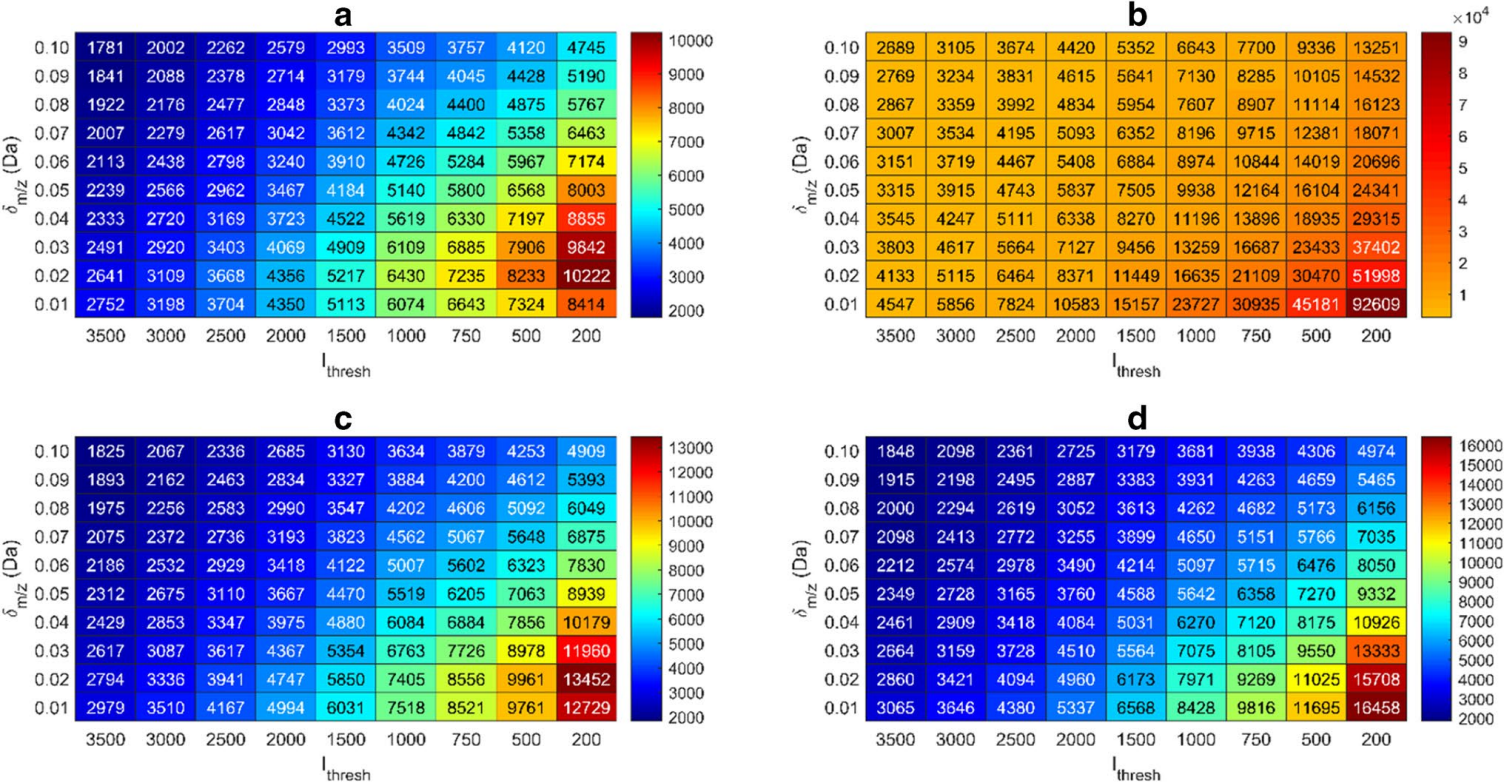
The number of *m/z* traces extracted from the raw data plotted against increasing *m/z* deviation (*δ*_*m/z*_) in Da and *I*_thresh_ using **a** the OMG and **b** the TGJ algorithm, respectively. In panels **c** and **d**, a *ρ*_gap allowed_ value of 1 and 2 was used for the OMG algorithm, respectively. The colours blue to brown represent increasing number of *m/z* traces. A value of 8 scans was used for ρ_min_. A value of *ρ*_gap allowed_ equal to zero was chosen for the OMG algorithm in panel **a**. See Fig. 1 step 3 for a visualisation of the *ρ*_min_ and *ρ*_gap allowed_

The key parameters requiring optimisation in the two ROI algorithms are *ρ*_min_, *I*_thresh_ and *δ*_*m/z*_. *I*_thresh_ must be set below the baseline of the least abundant peak of interest to ensure its inclusion (Fig. 1, step 3). The optimal *δ*_*m/z*_ was determined by testing values between 0.01 and 0.10 Da on seven injections of the wastewater effluent extracts onto the LC-HRMS. An increasing number of *m/z* traces were detected in the samples by the OMG algorithm as a function *δ*_*m/z*_ until 0.02 Da, after which the number of *m/z* traces with a unique *m/z* decreased (Fig. 2a, *ρ*_gap allowed_ equal to zero scans).

The mass spectral resolution was compromised at values of *δ*_*m/z*_ > 0.03 Da for this dataset (Fig. S1a-Fig. S2a). At these values, the mean and median ROI lengths exceeded the expected peak width, since adjacent ROIs in the *m/z* dimension, potentially originating from different chemical events, were merged into the same *m/z* trace. This led to higher variation within each *m/z* trace or ROI and increased *m/z* deviation due to the more heterogeneous collection of *m/z* measurements included in each trace (Fig. S4).

For *δ*_*m/z*_ values < 0.02 Da, potentially relevant ROIs were removed from the final data matrix. This occurred because splitting the *m*/*z* traces into multiple adjacent ROIs resulted in fewer consecutive scans than *ρ*_min_, causing their removal by the chromatographic filter. This effect was reflected in shorter mean and median ROI lengths and a reduced number of detected *m/z* traces (Fig. S1−2, Fig. 2). To address this issue, the parameter *ρ*_gap allowed_ was implemented. This parameter enables the retention of two ROIs separated by a user-defined number of scans, provided their combined length is at least *ρ*_min_. Generally, a *ρ*_gap allowed_ value of 1 or 2 yields higher number of detected compounds and longer ROIs especially for higher values of *I*_thresh_ and lower values of *δ*_*m/z*_ (Fig. S1−3). We also observed that more *m/z* traces were retained in the data matrix (Fig. 2a, c, d).

In the TGJ algorithm, gaps are addressed by performing a linear interpolation plus a random noise contribution between the two points on each side of the gap. All scan points for all *m/z* traces are consequently given an intensity value, which contrasts the sparse nature of HRMS data. No optimum for *δ*_*m/z*_ was observed for the TGJ algorithm, based on the number of *m/z* traces, nor the length of the ROIs, since the TGJ chromatographic filter did not require the mass spectral measurements to be in consecutive scans, as was the case for the OMG algorithm. Rather a minimum number of occurrences within an *m/z* trace was required. Therefore, the TGJ chromatographic filter was found to be less efficient in excluding ROIs that did not correspond to a real chromatographic peak (Fig. 2, Fig. S1 and Fig. S2b). As a result, more *m/z* traces were retained in the TGJ algorithm (Fig. 2), leading to a lower data compression rate.

In Fig. S2, the median ROI length was shown to range from 12 to 23 scans for the OMG algorithm, compared to a single scan for the TGJ algorithm, regardless of *I*_thresh_ and *δ*_*m/z*_. This indicates that the OMG algorithm effectively excluded ROIs shorter than a chromatographic peak. When ROIs with lengths below eight scans were excluded, the median ROI length in the TGJ algorithm increased to 11–14 scans, comparable to the OMG algorithm. The more efficient chromatographic filtering in the OMG algorithm suggests it could reduce false positive rate in feature detection and compound identification by filtering out small spikes (step 3, Fig. 1). Reducing false positives in NTS has been the focus of several previous publications, since it increases the reliability of NTS results along with reducing the time needed for manual exclusion of false positive ROIs [15, 17].

The maximum number of detected compounds for the OMG algorithm was 53, observed at *δ*_*m/z*_ > 0.06 Da and *I*_thresh_ ≤ 750 counts. The number of detected compounds decreased with decreasing *δ*_*m/z*_ and increasing *I*_thresh_ for both algorithms. However, the TGJ algorithm was less affected by these parameters with respect to the number of detected compounds. This suggests that the OMG algorithm is more sensitive to *I*_thresh_ and *δ*_*m/z*_ compared to the TGJ algorithm, which was explained by the fact that the risk that a ROI was excluded by the chromatographic filter increased as a consequence of having data points with too low intensity or a too heterogeneous collection of *m/z*’s compared to *δ*_*m/z*_.

To mitigate this, the parameter *ρ*_gap allowed_ can be adjusted. Unlike the TGJ algorithm, the OMG algorithm’s chromatographic filter aligns more closely with chromatographic peak width, making it easier for data analysts to select suitable values. The *m/z* deviation decreased with lower *δ*_*m/z*_ and higher *I*_thresh_ values (Fig. S4), since fewer data points were included in each *m/z* trace, reducing the *m/z* variance. The *m/z* deviations were similar between the two algorithms, with *ρ*_gap allowed_ having minimal impact (Fig. S4).

The proposed optimisation scheme is also believed to be applicable to the centWave algorithm, where consecutive mass spectral measurements are required [6]. Myers et al. [18] investigated key differences in feature detection between MZmine2 and XCMS but did not address the optimisation of *δ*_*m/z*_*.* As an alternative to ROI detection, Reuschenbach et al. [19, 20], have put forth a collection of algorithms named qAlgorithms, which uses probabilistic approach that is applicable to continuum HRMS data reducing subjectivity. If continuum data is not available, a user-defined *m/z* threshold is required to be optimised. In this case, it loses its advantages of being user-parameter-free.

In this study, an optimal *δ*_*m/z*_ value of 0.02 Da was identified. At this value, along with an *I*_thresh_ of 200, a ten-fold reduction in data size was achieved when storing the pre-processed files on disk. The same optimisation was applied to the OMG algorithm, specifying *δ*_*m/z*_ in ppm. We achieved a mean RMS-mDev of 9 ppm using a *δ*_*m/z*_ of 36 ppm (~0.02 Da at 500 *m/z*) and *I*_thresh_ of 200 compared to a mean RMS-mDev of 20 ppm when using a *δ*_*m/z*_ of 0.02 Da. The improvement with ppm specification was expected, since TOF mass spectrometry inherently maintains a constant ppm deviation across the *m/z* range [21]. This trend was evident in the data, where *m/z* deviations were higher for compounds with lower *m/z* values when *δ*_*m/z*_ was specified in Da.

Due to the chromatographic filter implemented in the TGJ algorithm, the presented *δ*_*m/z*_ optimisation could not be used; instead *δ*_*m/z*_ must be selected manually. Previous studies suggest setting *δ*_*m/z*_ as a multiple of the instruments mass resolution, emphasising the need for analysts to evaluate suitability for each dataset [8, 9]. However, this manual approach introduces variability in results, based on the data analyst’s choices with respect to the observed ROI length (Fig. S1−2), *m/z* deviation (Fig. S4), and the number of detected ROIs (Fig. 2).

### Scalability of the ROI algorithms and its implication to compound detection

**Fig. 3:**
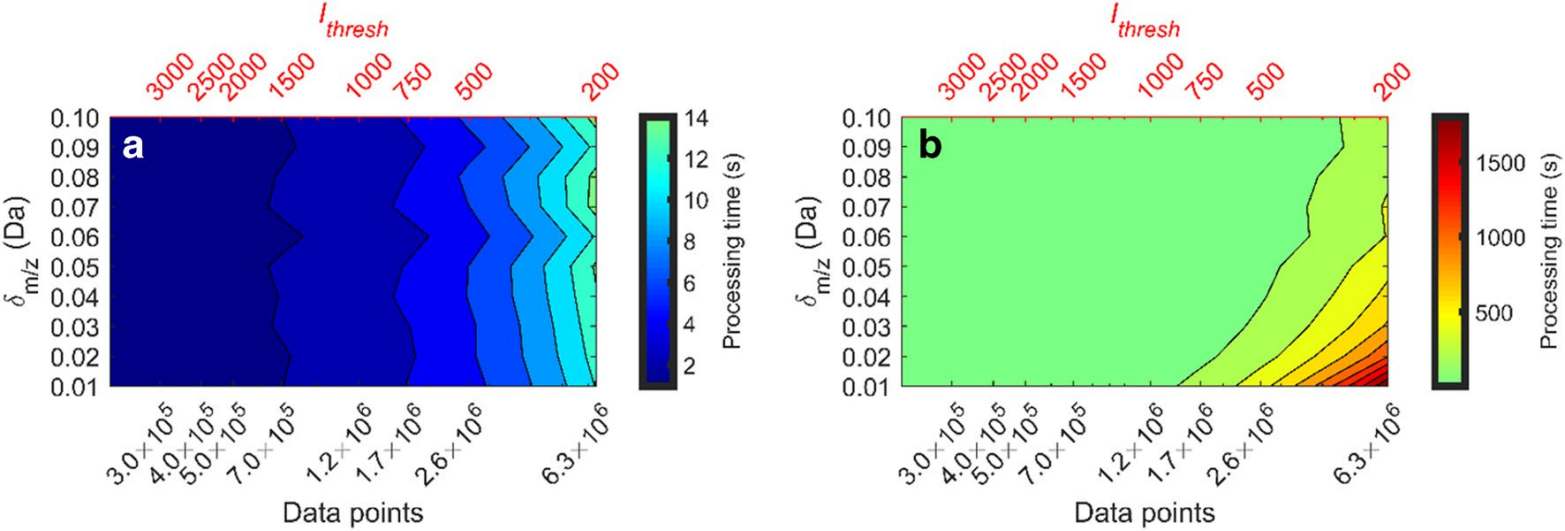
Mean processing time (in seconds) for seven replicate injections of the pooled wastewater effluent extract, plotted as a function of the *m/z* deviation (*δ*_*m/z*_) and noise threshold (*I*_thresh_, secondary *x*-axis) for the OMG (**a**) and TGJ (**b**) algorithms. The tested *δ*_*m/z*_ values and noise thresholds are shown in the figure. The primary *x*-axis indicates the mean number of data points remaining in the data file after excluding those with intensity < *I*_thresh_ (Fig. 1, steps 2 and 3). A value of *ρ*_gap allowed_ = 0 was used for the OMG algorithm

The maximum processing time for one sample was 15 s vs. 2.0×10^3^ s for the OMG and the TGJ algorithms, respectively (Fig. 3a and b). The improvement in processing time ranged from a factor 3 to 166 across the tested values of *δ*_*m/z*_ and *I*_thresh_. Figure 3a and b show that processing time for both algorithms increased as *I*_thresh_ decreased, with the TGJ algorithm being more sensitive to *δ*_*m/z*_. Specifically, for the TGJ algorithm, reducing *I*_thresh_ from 2000 to 200 caused processing time to increase by factors of 68 and 240 for *δ*_*m/z*_ values of 0.1 and 0.01, respectively. In comparison, the OMG algorithm showed smaller increases, with processing time rising by factors of 14 and 11 for the same *δ*_*m/z*_ values.

Since *I*_thresh_ was used as a proxy for file size, Fig. 3 shows that the TGJ algorithm’s processing time increased more than linearly with the number of data points, rising from 3.0×10^5^ to 6.3×10^6^, equivalent to a 21-fold increase in data size. In contrast, the OMG algorithm showed a sub-linear increase, with the slope of the relationship between processing time and the number of data points ranging from ~ 0.5 to 0.7 (< 1). For the TGJ algorithm, the slopes were significantly steeper, ranging from 3 to 11 for *δ*_*m/z*_ values of 0.1 and 0.01. Consequently, the difference in processing time between the OMG and TGJ algorithm increased with decreasing *δ*_*m/z*_ and *I*_thresh_*.*

The improved speed of the OMG algorithm can be attributed to several factors: primarily due to a more efficient *m/z* trace detection procedure (Fig. 1, step 2), and, to a lesser extent, to memory pre-allocation and footprint, lower computationally complexity and vectorised operations. These improvements allowed data analysts to optimise *δ*_*m/z*_ and *I*_thresh_ in multiple steps without major concerns about processing time. It is noteworthy that all *m/z*’s and intensities are required to be loaded into the memory by the sorting step by the OMG algorithm, whereas the TGJ could be modified to work scan-wise thereby reducing the memory footprint at the price of a higher computation time. However, this is not done in the tested implementation of the TGJ algorithm.

This flexibility is critical, as previous studies have recommended setting *I*_thresh_ at 0.25% of the most intense data point, which in this study corresponds to an *I*_thresh_ of ~7500 [9]. Applying this threshold would have resulted in only 36 of the 57 investigated compounds being detected due to insufficient data points per peak. The most intense signal in a chromatogram may not always be a peak relevant to the problem at hand; it could arise from blank contaminations, compounds in the washing phase or irrelevant sample components. Such signals can be orders of magnitude more intense than peaks of interest. Whilst a high *I*_thresh_ can reduce data compression time [22], its impact on the OMG algorithm is minimal: Even with the most conservative choices of *δ*_*m/z*_ (0.01) and *I*_thresh_ (200), the maximum processing time was approx. 15 s (Fig. 3). In contrast, other studies such as Dalmau, Bedia and Tauler [9] have used higher *I*_thresh_ potentially excluding trace-level compounds below the applied *I*_thresh_. Similarly, Schöneich et al. [23] demonstrated that noise threshold selection critically affects compound detection rates. Their study showed that at a spike level of 50 ppb, only 6–7 out of 18 spiked pesticides were detected using a noise threshold of 0.1%, whilst none was detected at 1 ppb. Our observations align with these findings: reducing *I*_thresh_ for the OMG algorithm increased number of detected compounds (Fig. S3). Lower *I*_thresh_ values preserved more data points at the edges of chromatographic peaks, ensuring the number of data points per peak met or exceeded *ρ*_min_. This improvement allowed the detection of more compounds, especially those at trace levels.

### Augmenting data matrices across samples

**Fig. 4:**
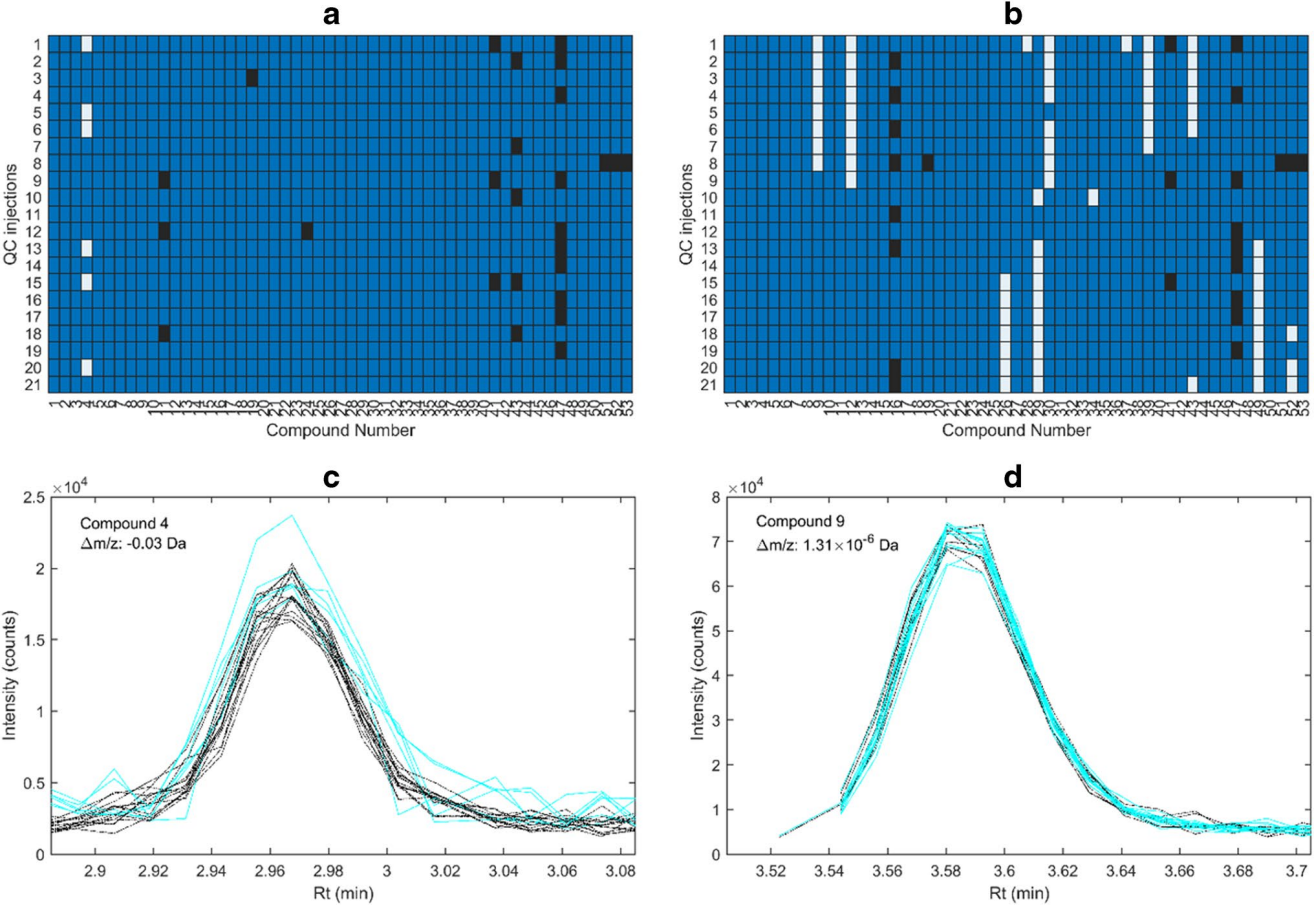
**a**, **b** Compounds detected in the same *m/z* trace in one sample as in the majority of the pooled wastewater extracts, i.e. quality control (QC) injection, are shown in blue for both OMG and TGJ algorithms. If a compound was detected in a different *m/z* trace compared to the majority, it is displayed in white. Compounds that were not detected are shown in black. **c** The elution profiles of theobromine (compound 4) across the 21 injections, highlighting a misclassification in the OMG algorithm where the compound was grouped into two separate *m*/*z* traces. These *m/z* traces are represented by cyan and black lines, with the Δ*m/z* values indicated on the plot. **d** A similar scenario for amisulpride (compound 9) using the TGJ algorithm, showing misclassification into two *m*/*z* traces. For the OMG algorithm, a *ρ*_gap allowed_ value of 0 was applied

The matrices for the 21 LC-HRMS chromatograms from the wastewater effluent sample were augmented (stacked row-wise) using *δ*_*m/z*_ = 0.02 for both algorithms. The *I*_thresh_ values were set at 200 counts for OMG and 3500 counts for TGJ. This resulted in a mean RMS-mDev of 17 ppm for OMG and 11 ppm for TGJ across the 21 injections. The lower RMS-mDev for TGJ was due to its higher noise threshold (Fig. S4). Both algorithms detected 53 compounds under these conditions (Fig. 4a, b).

In Fig. 4a, b, the OMG algorithm demonstrated a higher success rate in augmenting compounds, as a larger fraction of the 53 compounds had identical *m/z* values in the augmented data matrix. Using the OMG algorithm, only one compound in six samples (out of 53 compounds across 21 chromatograms) was wrongly augmented into different *m/z* traces. For TGJ, this occurred for 12 compounds across 19 samples.

The elution profiles of a misclassified compound are shown in Fig. 4c, d, clearly indicating that the profiles originate from the same compound. Ideally, all compounds should be grouped into the same *m/z* trace in the augmented matrix, resulting in identical *m/z* deviations across all samples. Deviations from this uniformity in RMS-mDev indicate misclassification, where one or more compounds were incorrectly grouped into separate *m/z* traces across replicates.

Misclassified compounds can be identified automatically by comparing the *m/z* values of each compound in each sample to the most prevalent *m*/*z* value for that compound across all 21 replicates (Eq. 3). Compounds with *m/z* values that deviate from this most prevalent value indicate unsuccessful grouping. This approach provides a systematic method for identifying and resolving misclassified *m/z* traces in augmented data matrices.

The high success rate of data augmentation in the OMG algorithm is pivotal for its effectiveness as a pre-processing step for curve-resolution methods with tri-linearity constraints. Misclassified *m/z* traces for a single compound would introduce non-rank-one contributions, undermining the trilinear structure. Similarly, in feature detection workflows, incorrect grouping of *m/z* traces would falsely increase the perceived complexity of the sample, leading to inaccurate results. The high success rate of grouping *m/z* traces serves as a good foundation for further grouping of adducts, fragments, and in-source fragments.

## Conclusion

This paper demonstrates that the OMG algorithm is a suitable tool for signal processing workflows in chromatographic data hyphenated to HRMS, particularly for detecting trace-level compounds. Compared to the state-of-the-art TGJ algorithm, OMG exhibits improved scalability, faster processing times, and higher compression rates, whilst maintaining high-quality ROIs for subsequent analysis.

An automated approach was developed for optimizing *δ*_*m/z*_ and validating the grouping of compounds across samples. The reduced reliance on manual parameter selection ensured consistent data processing and improved the grouping success rate; misclassification was limited to one compound in six samples for OMG compared to 12 compounds in 19 samples for TGJ.

Whilst OMG demonstrated strong performance, *m/z* deviations were still higher when compared to raw data inspection. Addressing this limitation could be a focus for future research to further enhance accuracy and usability.

## Supplementary Information

Below is the link to the electronic supplementary material.

Supplementary file1 (DOCX 1558 KB)

## Data Availability

Data is available through GitHub: https://github.com/OskarMunkKronik/regionofinterest.
