# Supplementary material for: A fast region of interest algorithm for efficient data compression and improved peak detection in high-resolution mass spectrometry
Source: Anal Bioanal Chem. 2025 Jan 9;417(27):6065–73. doi: 10.1007/s00216-024-05718-7 (PMC12583364; doi:10.1007/s00216-024-05718-7)
Supplement: Supplementary file 1 — Supplementary file1 (DOCX 1558 KB) [file 216_2024_5718_MOESM1_ESM.docx]

# Supplementary Information: A fast region-of-interest algorithm for efficient data compression and improved peak detection in high-resolution mass spectrometry

*Oskar Munk Kronik^*^, Jan H. Christensen, Nikoline Juul Nielsen, Selina Tisler, Giorgio Tomasi*

*Department of Plant and Environmental Science, University of Copenhagen, Thorvaldsensvej 40, Frederiksberg DK-1871, Denmark*

*^*^Corresponding author:* *E-mail address: omkr@plen.ku.dk (O.M. Kronik).*


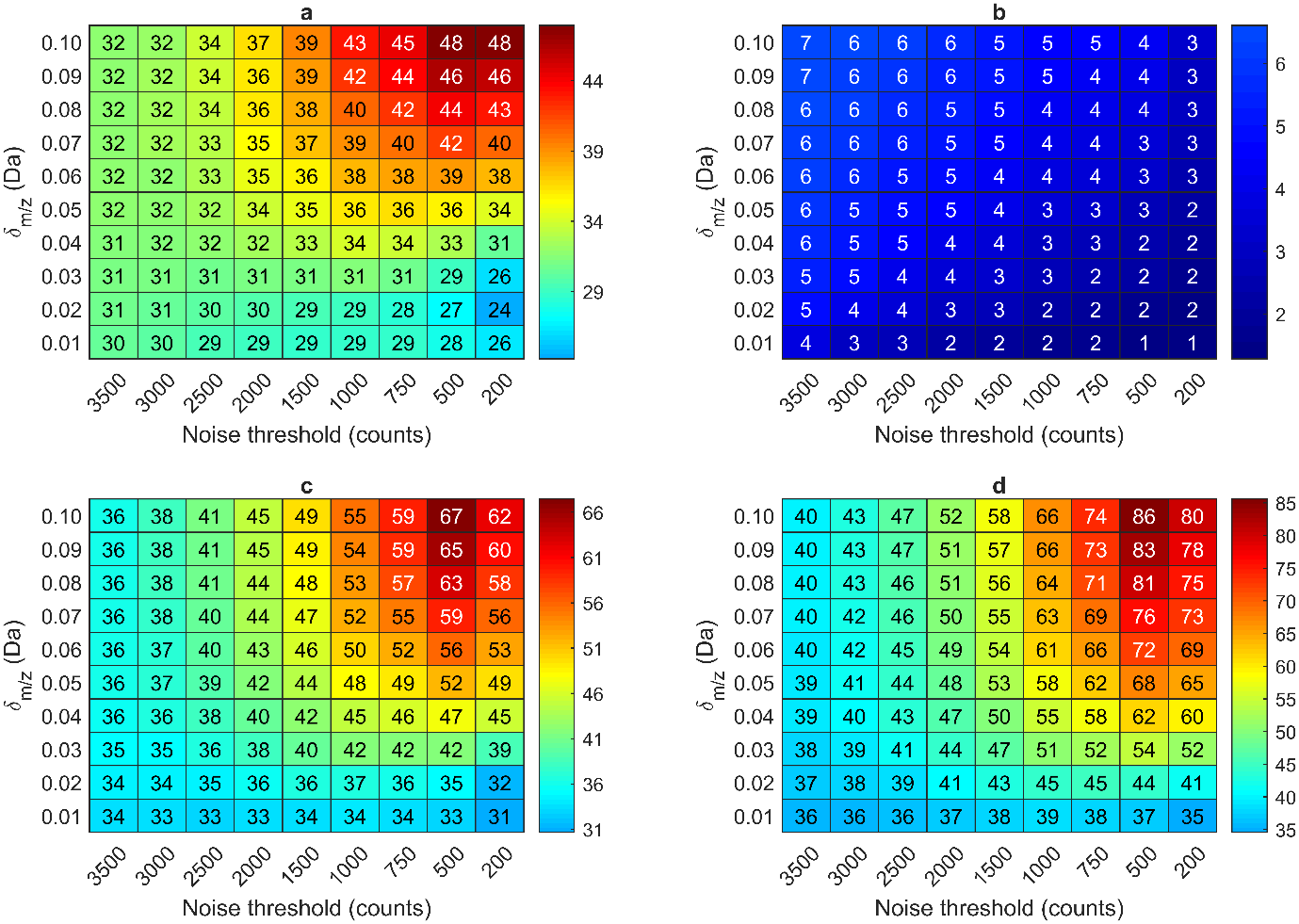


Fig. S1 The mean length of each ROI based on seven QC samples as a function of *δ_m/z_* and noise threshold using the a) OMG (*ρ_gap allowed_* = 0) and b) TGJ algorithm, c) OMG (*ρ_gap allowed_* = 1), and d) OMG (*ρ_gap allowed_* = 2), respectively


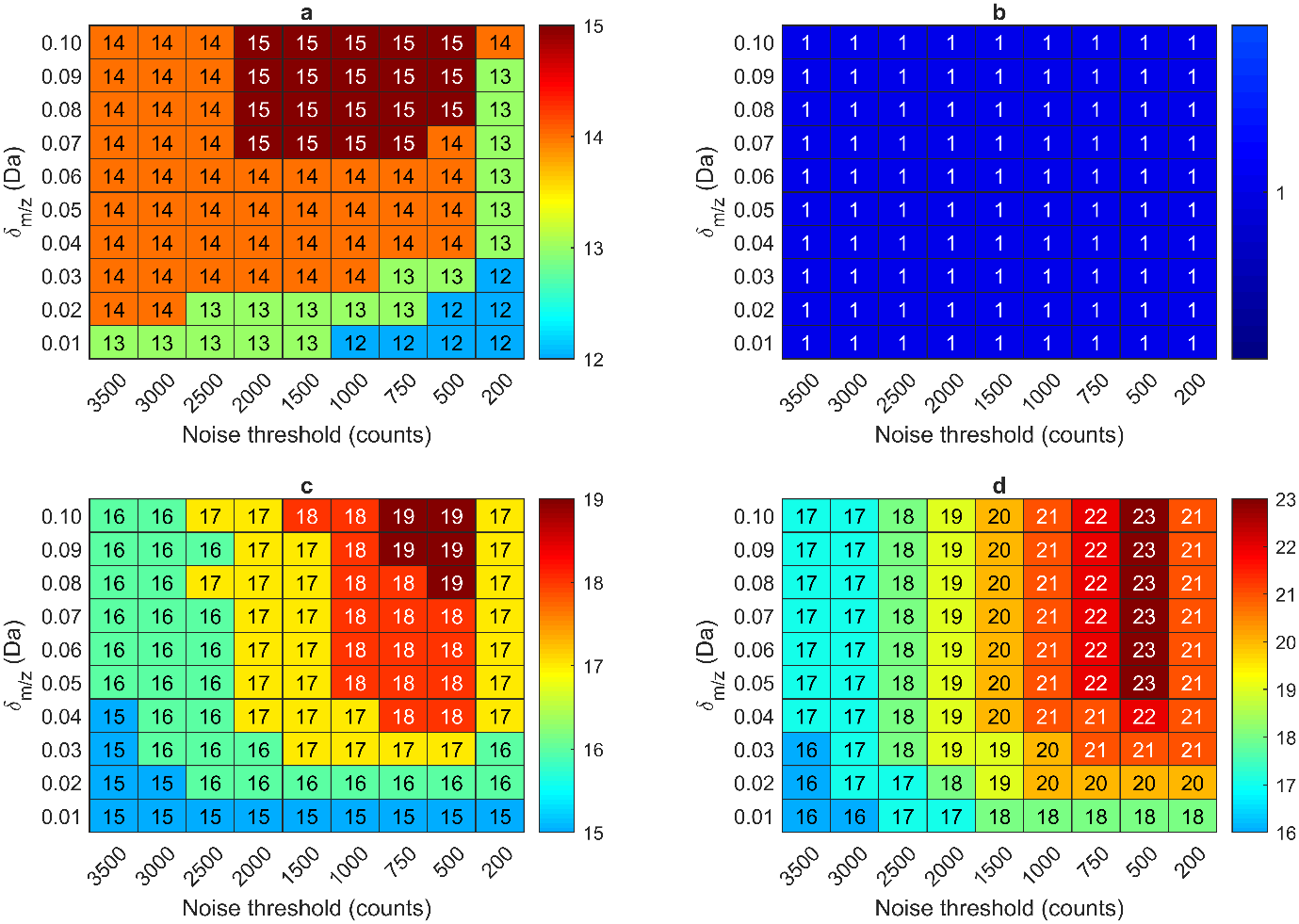


Fig. S2 The median length of each ROI based on seven QC samples as a function of *δ_m/z_* and noise threshold using the a) OMG and b) TGJ algorithm, c) OMG (*ρ_gap allowed_* = 1), and d) OMG (*ρ_gap allowed_* = 2), respectively


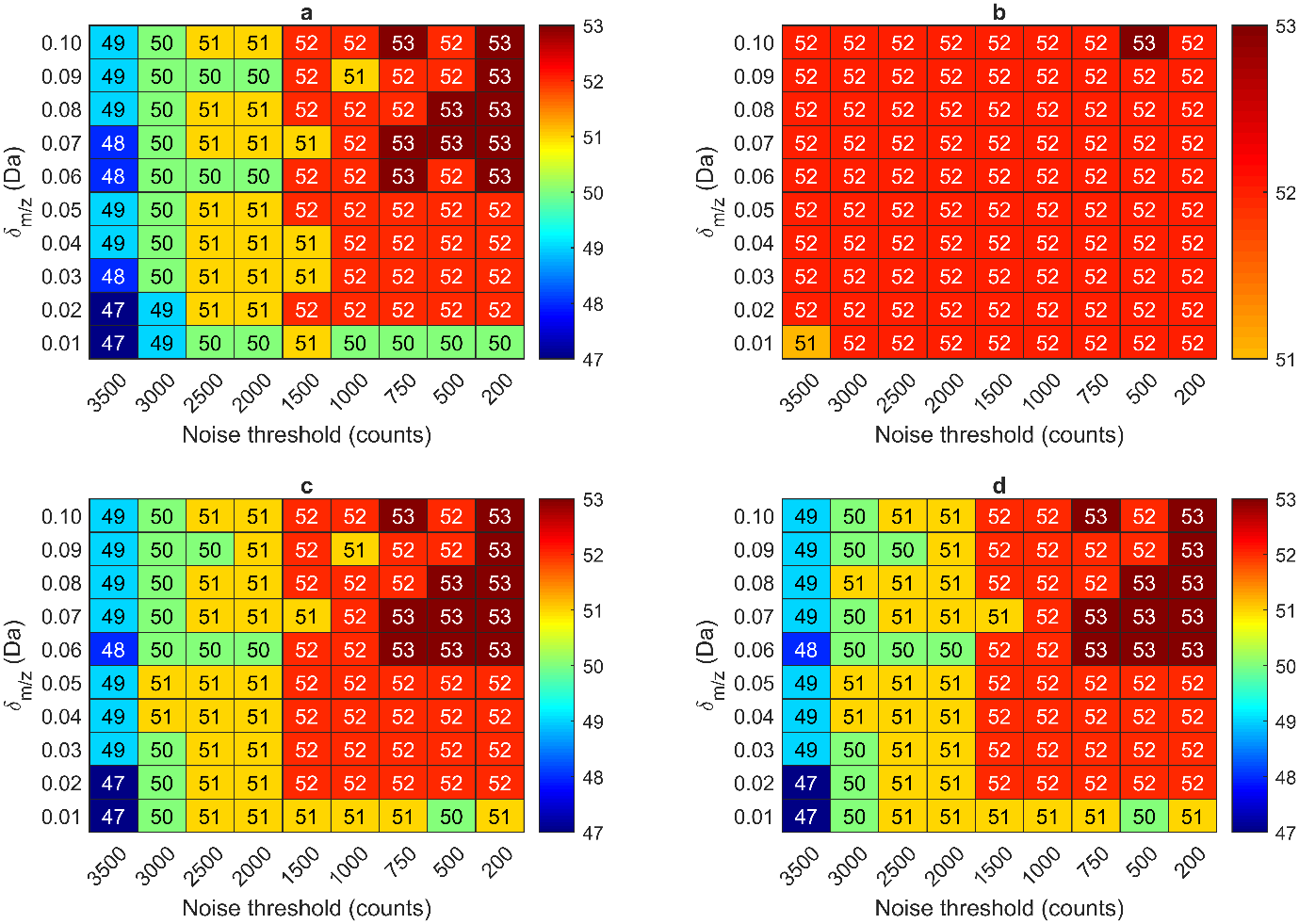


Fig. S3 The mean number of detected compounds in seven QC samples as a function of *δ_m/z_* and noise threshold using the a) OMG (*ρ_gap allowed_* = 0) and b) TGJ algorithm, c) OMG (*ρ_gap allowed_* = 1), and d) OMG (*ρ_gap allowed_ = 2*), respectively


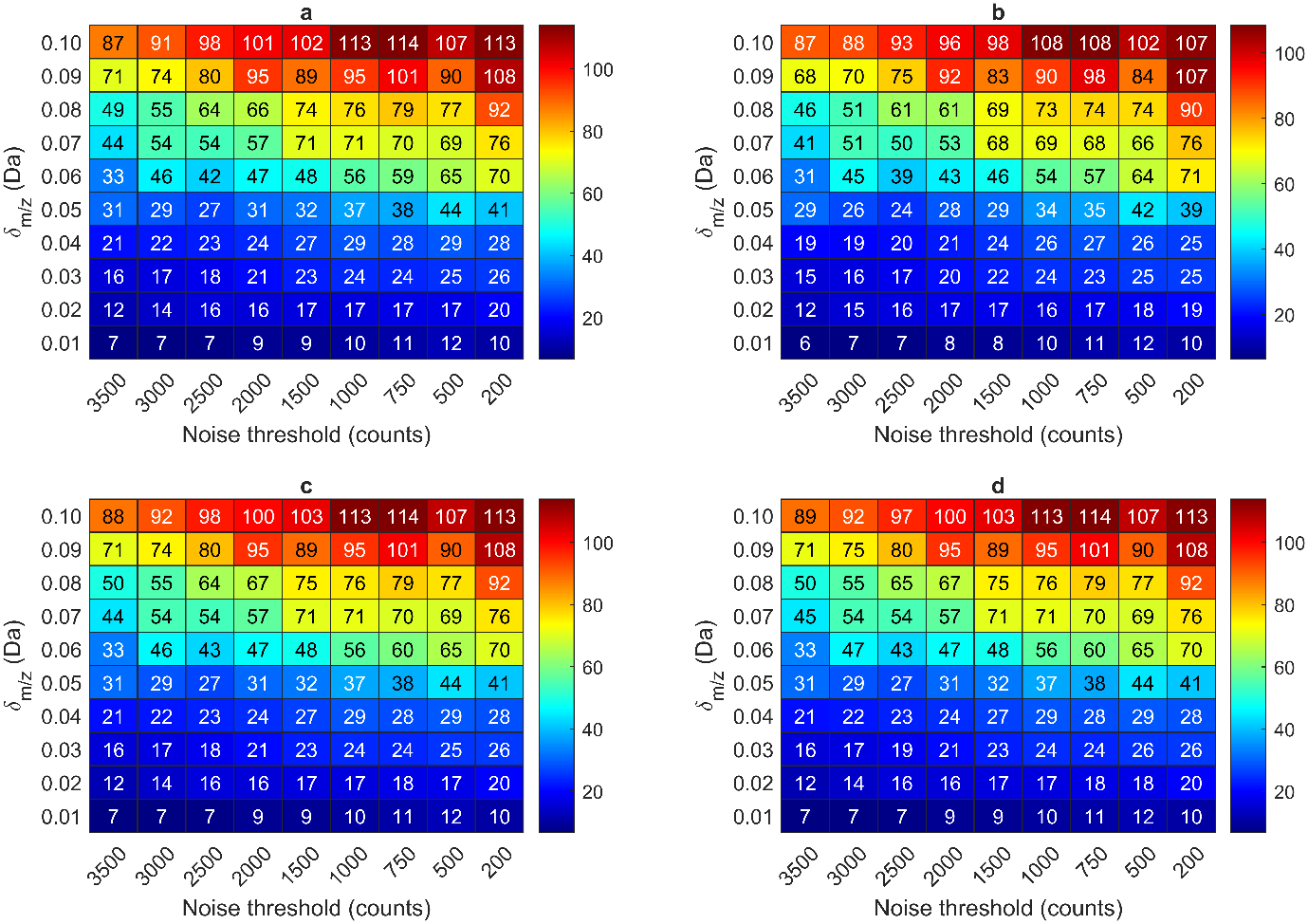


Fig. S4 a-b) The root-mean squared *m/z deviation* in ppm calculated from the number of detected compounds seen in Fig. S3. The mean was calculated from the seven QC samples plotted as a function of *m/z* deviation (*δ_m/z_*) and noise threshold using the a) OMG (*ρ_gap allowed_* = 0), b) TGJ algorithm, c) OMG (*ρ_gap allowed_* = 1), and d) OMG (*ρ_gap allowed_* = 2), respectively
